# Effect of methylene blue on hemodynamic and metabolic response in septic shock patients

**DOI:** 10.1097/MD.0000000000028599

**Published:** 2022-01-21

**Authors:** Fabio Luis-Silva, Mayra Gonçalves Menegueti, Corina dos Reis Sepeda, Bruno C. Petroski-Moraes, Lucas Sato, Leandro Moreira Peres, Christiane Becari, Anibal Basile-Filho, Paulo R.B. Evora, Olindo Assis Martins-Filho, Maria Auxiliadora-Martins

**Affiliations:** a Division of Intensive Care Medicine, Department of Surgery and Anatomy, Ribeirão Preto Medical School, University of São Paulo, Ribeirão Preto, Brazil; b Professor of Clinical Medicine, Barao de Maua University Center - Ribeirão Preto / São Paulo, Brazil; c Ribeirão Preto Nurse School, University of São Paulo, Ribeirão Preto, Brazil; d Division of Vascular and Endovascular Surgery, Department of Surgery and Anatomy, Ribeirão Preto Medical School, University of São Paulo, Ribeirão Preto, Brazil; e Division of Cardiac Surgery, Department of Surgery and Anatomy, Ribeirão Preto Medical School, University of São Paulo, Ribeirão Preto, Brazil; f René Rachou Institute, Oswaldo Cruz Foundation, FIOCRUZ-Minas, Belo Horizonte, Brazil.

**Keywords:** intensive care, methylene blue, septic shock, study protocol

## Abstract

**Introduction:**

Septic shock is a lethal disease responsible for a large proportion of deaths in the Intensive Care Unit (ICU), even with therapy centered on fluid resuscitation, use of vasopressors and empirical antibiotic therapy applied within the first hour of diagnosis. Considering the multifactorial pathophysiology of septic shock and the mechanism of action of vasopressors, some patients may not respond adequately, which can lead to the maintenance of vasodilatation, hypotension and increased morbidity, and mortality. This protocol aims to verify whether the use of methylene blue in septic patients with an early diagnosis can contribute to an earlier resolution of a shock compared to standard treatment.

**Methods and analysis:**

This is a study protocol for a single-center randomized clinical trial design in an ICU of a tertiary university hospital. In this study, we intend to include 64 patients aged between 18 and 80 years with a diagnosis of septic shock, of any etiology, with up to 72 hours of evolution after volume restoration, using norepinephrine at a dose ≥0.2 μg/kg/min and vasopressin at a dose of 0.04 IU/min. After the initial approach, we will randomize patients into two groups, standard care, and standard care plus methylene blue. The sample size was calculated in order to show 30% differences in septic shock resolution between groups. The Research Ethics Committee approved the study, and all patients included will sign an informed consent form (Clinical registration: RBR-96584w4).

## 1 Introduction

Sepsis is a serious medical condition defined as a life-threatening organ dysfunction caused by an unregulated host response to infection.^[1,2]^ Septic shock is defined as the need for vasopressors to maintain a mean arterial pressure ≥ 65 mm Hg associated with a serum lactate level >2 mmol/L (>18 mg/dL) after adequate fluid resuscitation. In this condition, profound cellular and metabolic abnormalities in the cardiovascular, pulmonary, renal, hematological, gastrointestinal, endocrine, and neurological systems can occur and are associated with an increased risk of mortality.^[3]^

Septic shock treatment protocols are well established around the world, and it is briefly composed of a triad that includes hemodynamic resuscitation with crystalloid, infusion of vasopressors and administration of antimicrobials within the first hour of diagnosis.^[4]^ Although this triad has been shown to reduce mortality,^[5–7]^ mortality rates from septic shock are still above 40%, which is a major health problem affecting millions of people around the world each year despite all the technological and scientific advancement.^[8]^

In this context, new therapeutic improvements are needed and are under study. In recent decades, several studies have been carried out on the mechanisms of nitric oxide and its important role as a vasodilator. Under physiological conditions, L-arginine is converted to nitric oxide by a constitutive isoform of nitric oxide synthase (cNOS).^[9,10]^ The nitric oxide produced freely diffuses through cell membranes and activates the second messenger guanylate cyclase, which converts guanosine triphosphate to cyclic guanosine monophosphate, resulting in vascular smooth muscle relaxation.^[11,12]^

In sepsis, some inflammatory mediators such as endotoxins and cytokines such as tumor necrosis factor alpha and interleukin 2 can induce a calcium-independent induced nitric oxide synthase (iNOS), leading to a sustained production of nitric oxide that does not respond to negative feedback mechanisms physiological.^[12,13]^ Thus, nitric oxide collaborates with significant vasodilatation and hypotension from septic shock, and its increased levels may be associated with decreased responsiveness to vasopressors therapy, altered regional blood flow distribution, increased capillary leakage, and multiple organ dysfunction.^[9–13]^

Some pilot studies and case reports have already shown the benefit of the association of methylene blue as a supporting agent in the treatment of vasodilatation resulting from septic shock because of its inhibitory action on nitric oxide-mediated vasodilatation^[13]^ However, most of these studies were not carried out in the first hours of septic shock, but when the patient had already undergone a series of interventions and the shock had already been installed for a long period.^[14–18]^ The aim of this protocol is to use methylene blue in the first 72 hours of septic shock diagnosis and to verify if its use can contribute to an earlier resolution of the shock compared to standard treatment.

## 2 Patients and methods

### 2.1 Study participants and eligibility criteria

The inclusion criteria for this study are: patients aged 18 to 80 years, of both genders, admitted to the ICU within the first 72 hours after the diagnosis of septic shock, as established by the consensus published in 2016 and 2021,^[2,5]^ which includes suspected infection with two or more points on the SOFA score associated with the need to use vasopressors to maintain mean arterial pressure (MAP) ≥65 mm Hg and lactate ≥2.0 mmol/L after adequate fluid resuscitation using norepinephrine in dose ≥0.2 μg/kg/min and vasopressin at a maximum dose for septic shock of 0.04 IU/min.

The Simplified Acute Physiology Score (SAPS-3)^[19,20]^ severity score will apply to all included patients. Exclusion criteria are: pregnant or postpartum women, previous septic shock in the same hospital, patients in palliative care, patients undergoing chemotherapy or using serotonergic drugs, monoamine oxidase or linezolid inhibitors, acute respiratory distress syndrome (ARDS), deprived persons of freedom and imminent death during the protocol.

### 2.2 Randomization and allocation

The randomization process will be carried out using digital software by a person not directly linked to the patient's treatment. The study is not blinded because of the bluish coloration of the skin and secretions from the use of methylene blue, and there is no innocuous compound that can be used with similar effect for the standard treatment group. Once the study team confirms that a subject meets all study entry criteria, they will be randomized to one of the study groups. All patients will receive a copy of the informed consent form to take part in the study, besides the verbal explanation of the step-by-step study.

### 2.3 Study procedures

#### 2.3.1 Primary outcome

The primary endpoint will be the quantification of the norepinephrine dose in the group that will receive the methylene blue group and standard care compared to the group that followed only the standard care. The outcome will be evaluated in both study groups, 24 and 48 hours after the initial assessment (since patients will use methylene blue for 48 hours). Sequentially, the control measurement will be performed 24 hours after the removal of the methylene blue (72 hours from the beginning of the protocol).

#### 2.3.2 Secondary outcome

The secondary outcome will include the comparison of the values of inflammatory mediators Interleukin-6, Interleukin-8, TNF-Alpha, TGF-Beta and the formation of plasma NO by the quantification of nitrite and nitrate in the intervention and standard treatment groups, during the evaluation periods in both groups.

### 2.4 Intervention protocol

**Figure 1:**
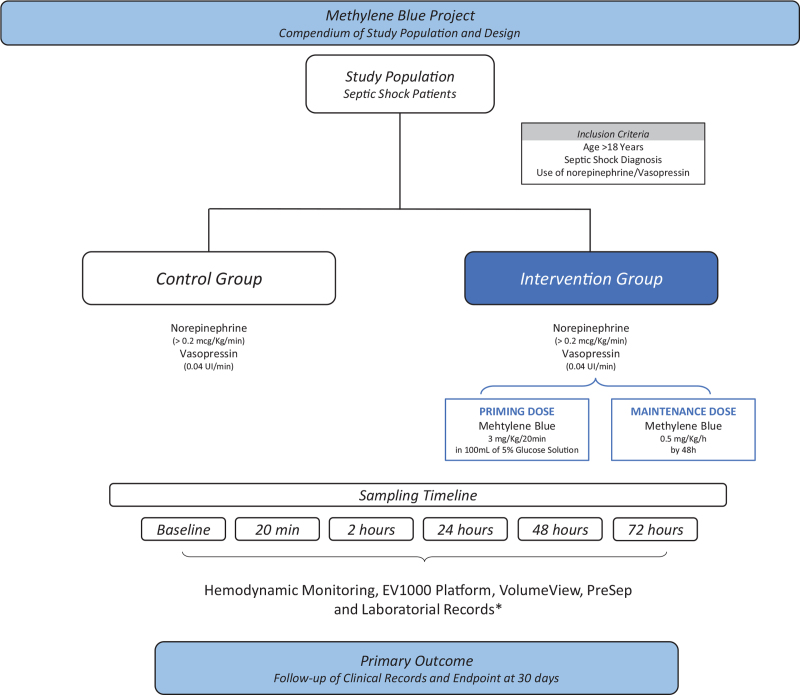
Compendium of study population and design.

Eligible patients were randomized to one of two groups, standard care plus methylene blue (Intervention group) or standard care (Control group) (Fig. 1). Standard care will include fluid resuscitation with crystalloids, use of vasopressors, and use of empirical antibiotics as per institutional protocol within the first hour of diagnosis.

All patients will have their weight measured using a bed scale (Styker or Eleganza brand) and compliance using an anthropometric measuring tape to calculate body surface area. Afterwards, a catheter will be inserted in the femoral vein (VolumeView System, Edwards Lifesciences Corporation) and central venous access in the jugular or subclavian vein (Presep, Edwards Lifesciences Corporation) for hemodynamic monitoring with the EV 1000 platform.

The initial collection of laboratory tests will be carried out (central venous and arterial blood gases, arterial lactate, bilirubin, complete blood count, sodium, potassium, urea, creatinine, and sample collection for dosage of inflammatory mediators in tubes with EDTA and heparin) followed by calibration and initial measurement of hemodynamic variables in all patients. The variables collected will be: cardiac index (CI), cardiac output (CO), mean arterial pressure (MAP), heart rate (HR), central venous pressure (PVC), systolic volume variation (SVV), systolic volume index (IVSi), pulmonary vascular permeability index (IPVP), global ejection fraction (EFG), global end-diastolic volume index (IDFG), pulmonary extravascular water index (IAPF), and systemic vascular resistance index (IRVSi).

The processing of samples for measurement of inflammatory mediators will include two steps, the first will be centrifugation at 3500 rpm for 10 minutes at 10°C and the second will be the collection of plasma with storage in Eppendorf tubes, identified and frozen at −70°C. After the initial measurement (baseline) of hemodynamic parameters, patients randomized to the intervention group will receive standard treatment associated with methylene blue at a dose of 3 mg/kg in 20 minutes and after 0.5 mg/kg/h for 48 hours and patients in the control group will receive standard treatment which will include fluid resuscitation, use of vasopressors and broad-spectrum antibiotics, which will be further adjusted according to the results of cultures. The protocol will continue with new measurements of the EV 1000 and collection of exams in the following times: 20 minutes (end of an attack dose of methylene blue), 2 hours, and 24 hours. In 48 hours, we will perform a new collection of exams and measurements of hemodynamic variables. After completion of measurements, the use of methylene blue will be suspended and after 72 hours, a new measurement of hemodynamic variables will be performed in both groups. After completion of the protocol, patients will be followed to assess the outcome for 30 days.

### 2.5 Other

All professionals will be trained to perform central venous puncture to install the PreSep catheter and puncture the femoral artery to insert the VolumeView, as well as to confirm the position of the PreSep catheter on the chest x-ray. The laboratory analyzes will be carried out by a trained professional and with double checking of the material (test and counter-test) to guarantee the quality of the evaluated data.

### 2.6 Statistical analysis

Statistical analysis will be performed using Stata SE version 14.0 (College station, TX) software. Initially, data will be described using median, maximum, and minimum values for quantitative variables and numbers and percentages for qualitative variables. To compare the doses of vasopressors used between groups, the Mann–Whitney test for independent samples will be used. To compare the variation in vasopressor doses at different times in the two groups, the Wilcoxon test for paired samples will be used.

### 2.7 Data management

Data will be collected directly from the monitoring device and will be stored to ensure reliability.

All participants will receive a numerical identification of the study itself. Personal data will be stored in the hospital's digital system, protected by a password to ensure confidentiality.

The responsible researcher will communicate any modifications or side effects to REBEC.gov and to the Research Ethics Committee and clinical research unit of the Hospital das Clínicas of the Faculty of Medicine of Ribeirão Preto.

All principal investigators will have access to the final trial dataset.

## 3 Discussion

Septic shock is a lethal disease responsible for a large proportion of deaths in the ICU even with the therapies currently applied, centered on fluid resuscitation, use of vasopressors and early empirical antibiotic therapy.^[15–18]^ Considering the multifactorial pathophysiology of septic shock and the mechanism of action of vasopressors, some patients may not respond adequately, which can lead to the maintenance of vasodilatation, hypotension, and morbidity and mortality The cause of this high morbidity and mortality depends on the virulence of the infectious agent and the intrinsic immunity of the host, the sustained organic dysfunction secondary to tissue hypoperfusion, besides the late initiation of treatment, which can significantly worsen the patient's prognosis.

The intention of this protocol is the insertion of an adjuvant therapy to control the hemodynamic condition to maintain hemodynamic stability until the etiological treatment is carried out. In this context, methylene blue becomes very interesting for being a medication used since the nineteenth century with proven hemodynamic effects since 1976, besides showing safety associated with minimal side effects when used in adequate doses.^[21–24]^ Methylene blue is widely used for reversal of vasoplegia in the postoperative period of major surgeries with excellent results.^[25–29]^ Our primary aim with this protocol is to identify whether methylene blue can become an effective, cheap and easily accessible alternative to treat septic shock in association with the conventional treatment currently used. Our hypothesis is that methylene blue may contribute to reducing vasopressor infusion, reversing refractory vasodilatation, improve hemodynamic and tissue perfusion, delay nitric oxide-induced mitochondrial death, besides being a low-cost medication, few side effects and easily accessible in all health units.

## Author contributions

MAM, OAMF, PRE, BC and ABF designed/revised the study protocol and applied for fundings.

**Conceptualization:** Mayra Gonçalves Menegueti, Olindo Assis Martins-Filho, Maria Auxiliadora-Martins.

**Formal analysis:** Mayra Gonçalves Menegueti.

**Funding acquisition:** Maria Auxiliadora-Martins.

**Methodology:** Mayra Gonçalves Menegueti, Olindo Assis Martins-Filho.

**Project administration:** Fabio Luis-Silva, Corina dos Reis Sepeda, Bruno C Petroski-Moraes, Lucas Sato, Leandro Moreira Peres.

**Resources:** Christiane Becari, Anibal Basile-Filho, Paulo R. Evora.

**Supervision:** Maria Auxiliadora-Martins.

**Writing – original draft:** Fabio Luis-Silva.

**Writing – review & editing:** Olindo Assis Martins-Filho, Maria Auxiliadora-Martins.
